# The association between sex hormones and prevalence of OA in US adults

**DOI:** 10.3389/fmed.2024.1425210

**Published:** 2024-12-12

**Authors:** Xiaoyuan Tian, Bocheng Zhang

**Affiliations:** Second Affiliated Hospital, Dalian Medical University, Dalian, China

**Keywords:** osteoarthritis, testosterone, estradiol, risk factors, hormones

## Abstract

**Background:**

Age and gender are commonly recognized as risk factors for osteoarthritis (OA), implying a potential association between sex hormones and OA pathogenesis. However, the precise role of sex hormones in OA remains elusive. Meanwhile, testosterone to estradiol (TT/E2) ratio is a new biomarker of sex hormone milieu. In this study, we aimed to investigate the relationship between sex hormones, specifically TT/E2 ratio, and the prevalence of OA among adults in the United States.

**Methods:**

This study is a cross-sectional study, and the data sourced from the National Health and Nutrition Examination Survey (NHANES) 2013–2016 cycles. This study primarily focuses on individuals aged 50 and above, employing multivariable logistic regression to examine the association between sex hormones and OA.

**Results:**

We included 2,615 participants (972 females). No significant association was observed between testosterone or estradiol levels and OA prevalence when analyzed separately. However, the TT/E2 ratio exhibited a robust inverse association with OA, particularly in females (adjusted OR = 0.61, 95% CI: 0.41–0.91, *p* = 0.02). A nonlinear relationship was observed in females, with a threshold effect indicating reduced OA risk when the TT/E2 ratio exceeded 0.3.

**Conclusion:**

The TT/E2 ratio was inversely associated with OA risk, with a stronger and more consistent effect observed in females. These findings suggest the TT/E2 ratio as a potential biomarker for OA risk stratification, particularly in postmenopausal females. Future longitudinal studies are warranted to clarify the causal role of the TT/E2 ratio in OA.

## 1 Introduction

Osteoarthritis (OA) is the most common bone and joint disease in the middle-aged and older adult, affecting approximately 654 million people worldwide (1). Due to the combined effects of an aging global population and increasing obesity, OA is becoming more common and imposes a significant economic burden on society (2). OA primarily involves the progressive degeneration of articular cartilage, accompanied by bone overgrowth (osteophytes), inflammation of the synovial membrane, and thickening or sclerosis of the subchondral bone. However, the specific mechanism of OA pathogenesis is still unclear, so there is a lack of effective treatments. Currently, non-steroidal anti-inflammatory drugs (NSAIDs) are the first-line treatment for OA; however, their efficacy diminishes in advanced stages of the disease and they also pose an increased risk of cardiovascular events (3). Despite the preventive potential of lifestyle modifications such as weight loss and appropriate exercise, further efforts are required to elucidate the risk factors and enhance OA prevention strategies (4).

Age is currently a recognized risk factor, and the prevalence increases significantly after the age of 50. In addition to age, obesity, sex factors, trauma and metabolic factors are closely related to the occurrence of OA (5). Compared with males, OA is more common in females, especially in postmenopausal females. This phenomenon suggests that changes in hormones in circulation or tissues, especially sex hormone levels, are closely related to OA (6). At present, some studies have investigated the relationship between sex hormones and OA, but there is still no clear conclusion.

Previous studies investigating the relationship between sex hormones and OA have produced variable results, often limited by sample size and scope. For instance, a randomized controlled trial with 200 participants assessed the influence of serum progesterone, estradiol, and testosterone on synovitis, structural changes, and pain severity in OA patients through magnetic resonance imaging (7). This study reported significant associations between these hormones and OA-related structural and symptomatic changes, with sex-specific variations, particularly more pronounced effects in female participants. However, its small sample size limits the statistical power and generalizability of its findings. Additionally, the trial did not address potential long-term hormone fluctuations or include diverse population samples (7). Further supporting the role of sex hormones in OA, a meta-analysis of 38 studies examining menopausal animal models highlighted the exacerbation of cartilage damage under low estrogen conditions, suggesting potential benefits of early estrogen intervention in reducing OA severity. Nevertheless, these findings are based on animal models that may not fully capture the complexity of human menopausal physiology, as the models primarily involve younger animals (8).

In addition to testosterone and estradiol, recent studies have found that the total testosterone to estradiol (TT/E2) ratio can better reflect the relationship between hormones and diseases, such as predictive biomarkers of cardiovascular disease (9) and mortality (10). However, there are currently no studies on TT/E2 ratio and OA. Whether the TT/E2 ratio reflects OA severity or serves as a predictive biomarker is unknown. In this study, we used the National Health and Nutrition Examination Survey (NHANES) database to analyze the relationship between sex hormones (including total testosterone, estradiol, and TT/E2 ratio) and prevalence of OA in people over 50 years old.

## 2 Materials and methods

### 2.1 Study design

The NHANES program evaluates the health and nutritional status of adults and children in the United States through interviews and physical examinations, which is conducted by the Centers for Disease Control and Prevention (CDC).^1^ The program selects a nationally representative sample of approximately 10,000 people every 2 years using a complex, stratified, multistage probability cluster sampling design. The data used in this study are from 2013 to 2016 cycles. In the NHANES, only these two cycles were analyzed simultaneously for testosterone and estradiol content in serum through isotope dilution liquid chromatography tandem mass spectrometry (ID-LC–MS/MS) method. Since sex hormone levels vary greatly between genders, we separately analyzed the relationship between sex hormones and OA in males or females. The study primarily focused on individuals aged 50 and above, as the prevalence of OA significantly rises after this age while primary degenerative OA is uncommon before 50 years old. We excluded missing self-reported arthritis data, other arthritis types, individuals with missing data on sex hormones as well as those with incomplete confounding factor records. Because hormone replacement therapy (HRT) may affect sex hormone levels, we excluded individuals who had received HRT. The survey was approved by the National Center for Health Statistics (NCHS) Ethics Review Board and all adult participants signed informed consent.

### 2.2 Diagnosis of OA

The diagnosis of OA was based on self-reported OA, and research has demonstrated that self-reported OA aligns with clinical diagnosis of OA in 81% of cases (11). These self-reports are personal interview data, conducted at home by trained interviewers using the Computer Assisted Personal Interview (CAPI) system, and the questionnaire can be found in Medical Conditions Questionnaire (MCQ). During the family interview, the interviewer will ask the participants a series of questions, such as “Has a doctor or other health professional ever told you that you have arthritis?” and “Which type of arthritis was it?” We regarded as OA who answered “Osteoarthritis or degenerative arthritis” to the question. Those who answered “Rheumatoid arthritis,” “Psoriatic arthritis,” “Other,” “Do not know” or refused to answer were eliminated.

### 2.3 Sex hormones

Sex hormones, as independent variables, mainly include testosterone and estradiol. Serum samples are collected on mobile examination centers (MECs) and stored at −20°C until shipped to the laboratory for concentration determination through ID-LC-MS/MS method. The lower limit of detection (LLOD) for testosterone is 0.75 ng/mL and for estradiol is 2.994 pg/mL. The value of results below the LLOD is the lower limit of detection divided by the square root of 2. The TT/E2 ratio is calculated using the formula total testosterone/(10 × estradiol) (12, 13).

### 2.4 Definition of covariates

Covariates mainly include demographic data, lifestyle and metabolic factors. We included age and race/ethnicity to adjust for differences in demographic data. The race/ethnicity variable was categorized as non-Hispanic White, non-Hispanic Black, Mexican American, and other races. We mainly use the smoking and alcohol state to correct lifestyle differences. The smoking state is defined according to the number of smoking as: never (smoked less than 100 cigarettes in life), former (smoked more than 100 cigarettes in life and smoke not at all now), now (smoked more than 100 cigarettes in life and smoke some days or every day). The alcohol status is defined as: never (had <12 drinks in lifetime), former (had ≥12 drinks in 1 year and did not drink last year, or did not drink last year but drank ≥12 drinks in lifetime), mild (1 drink per day for female and 2 drinks per day for male), moderate (2 drinks per day for female, 3 drinks per day for male or binge drinking ≥2 days per month), heavy (≥3 drinks per day for female, ≥4 drinks per day for male or binge drinking ≥5 days per month). Metabolic risk factors include hypertension, diabetes and body mass index (BMI). Sex hormone-binding globulin (SHBG) is the carrier of transported sex hormones, combined with most testosterone and estradiol in the cycle. In order to explore whether SHBG will interfere with the role of sex hormones in OA, we have also used SHBG as a covariate. This study focused on individuals aged 50 and older, which includes a mix of premenopausal and postmenopausal females. Given the significant impact of menopausal status on sex hormone levels, menopausal status was included as a confounding variable for females.

### 2.5 Statistical analyses

All statistical analyses were conducted by R software (version 4.3.1). We accounted for the complex sampling design and used MEC exam weight in all analyses to generate estimates generalizable to the 2013–2016 US non-institutional population. Baseline characteristics were compared using T test for continuous variables and chi-square test for categorical variables. We have established six multivariable logistic regression models between sex hormones and OA: crude model, no covariates were adjusted; Model 1: age and race were adjusted; Model 2: age, race, smoking, and alcohol state were adjusted; Model 3: age, race, smoking, alcohol state, hypertension, and diabetes were adjusted; Model 4: age, race, smoking, alcohol state, hypertension, diabetes, and BMI were adjusted. Model 5: age, race, smoking, alcohol state, hypertension, diabetes, BMI, and SHBG were adjusted. In female individuals, we established Model 6: age, race, smoking, alcohol state, hypertension, diabetes, BMI, SHBG, and menopausal status were adjusted. Furthermore, we employed a restricted cubic spline (RCS) model with knots placed at the 10th, 50th, and 90th percentiles to assess any nonlinear associations and dose–response trends between sex hormones and OA risk. To evaluate the nomogram model’s ability to discriminate OA risk, we used receiver operating characteristic (ROC) curve analysis. *p*-value less than 0.05 is considered to have statistical difference.

## 3 Results

### 3.1 Baseline and demographic characteristics

**Figure 1:**
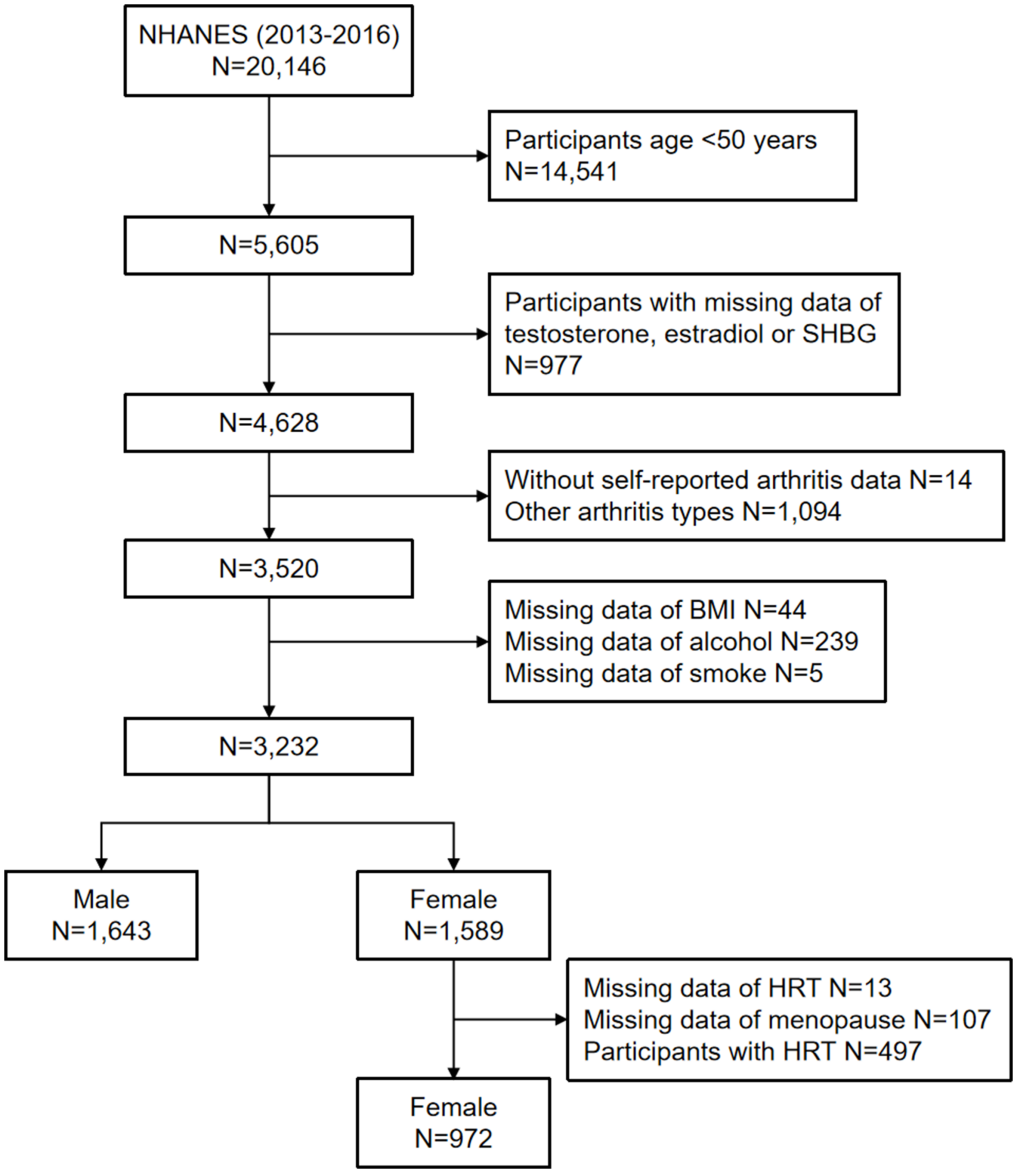
Flow chart of the selection of the study participants. NHANES, National Health and Nutrition Examination Survey; SHBG, sex hormone-binding globulin; BMI, body mass index; HRT, hormone replacement therapy.

The flow chart is shown in Figure 1. From 2013 to 2016, NHANES sampled a total of 20,146 people, weighted to represent 313,842,630 US citizens. Initially, 14,541 (72.2%) individuals under the age of 50 were excluded from the total sample of 20,146. Subsequently, those with missing sex hormones data (*n* = 977) were excluded, resulting in the inclusion of 4,628 representative individuals. Finally, individuals with missing self-reported arthritis data, other arthritis types and missing covariates were excluded. Among the 4,628 participants, 2,615 representative individuals meeting the inclusion criteria were included in this study, including 972 females and 1,643 males.

There are differences in age, race, BMI, hypertension and TT/E2 ratio between OA and non-OA groups, regardless of male or female. Compared with non-OA group, age and BMI were higher in the OA group. In OA participants, TT/E2 decreased significantly. There was a significant difference in estradiol only among females, with lower estradiol levels in the OA group. There was a significant difference in testosterone only among males, with lower testosterone levels in the OA group. Baseline characteristics are presented in Table 1.

**Table 1:** Characteristics of the study population.

	Female				Male			
Variable	Total (*n* = 972)	No (*n* = 683)	OA (*n* = 289)	*p*-value	Total (*n* = 1,643)	No (*n* = 1,341)	OA (*n* = 302)	*p*-value
Cycle				0.49				0.89
2013–2014, *n* (%)	426 (41.26)	299 (40.27)	127 (43.04)		785 (48.4)	631 (48.2)	154 (48.9)	
2015–2016, *n* (%)	546 (58.74)	384 (59.73)	162 (56.96)		858 (51.6)	710 (51.8)	148 (51.1)	
Age (yr), mean (SE)	63.86 (0.44)	62.37 (0.50)	66.58 (0.71)	<0.0001	62.82 (0.31)	62.20 (0.31)	64.87 (0.66)	<0.001
50–64, *n* (%)	546 (58.68)	424 (65.44)	122 (46.35)		891 (60.2)	763 (63.6)	128 (48.8)	
65~, *n* (%)	426 (41.32)	259 (34.56)	167 (53.65)		752 (39.8)	578 (36.4)	174 (51.2)	
Race				<0.0001				<0.0001
Mexican American, *n* (%)	172 (6.63)	135 (8.16)	37 (3.86)		247 (5.9)	213 (6.8)	34 (3.0)	
Non-Hispanic Black, *n* (%)	186 (11.10)	129 (12.00)	57 (9.44)		315 (8.4)	277 (9.3)	38 (4.4)	
Non-Hispanic White, *n* (%)	342 (68.90)	200 (63.00)	142 (79.66)		692 (74.4)	509 (71.3)	183 (84.8)	
Other, *n* (%)	272 (13.37)	219 (16.85)	53 (7.04)		389 (11.5)	342 (12.6)	47 (7.7)	
Marital status				0.05				0.04
Married or living with partner, *n* (%)	497 (55.90)	371 (59.86)	126 (48.69)		1,157 (75.3)	923 (73.5)	234 (81.3)	
Single, *n* (%)	475 (44.10)	312 (40.14)	163 (51.31)		486 (24.7)	418 (26.5)	68 (18.7)	
Educational level				0.09				0.06
Above high school, *n* (%)	457 (59.54)	299 (55.92)	158 (66.12)		856 (64.5)	673 (62.4)	183 (71.5)	
Below high school, *n* (%)	283 (18.01)	212 (19.72)	71 (14.90)		430 (15.0)	374 (16.1)	56 (11.2)	
High school or equivalent, *n* (%)	232 (22.45)	172 (24.36)	60 (18.98)		357 (20.5)	294 (21.5)	63 (17.3)	
PIR				0.73				0.16
Low income, *n* (%)	298 (20.80)	209 (23.53)	89 (21.41)		439 (16.0)	376 (18.7)	63 (13.1)	
Middle income, *n* (%)	338 (34.19)	243 (36.32)	95 (39.39)		536 (28.6)	436 (31.2)	100 (31.3)	
High income, *n* (%)	223 (36.38)	148 (40.16)	75 (39.20)		514 (47.1)	398 (50.1)	116 (55.6)	
BMI (kg/m^2^), mean (SE)	29.89 (0.31)	28.88 (0.37)	31.72 (0.43)	<0.0001	28.91 (0.17)	28.27 (0.23)	31.01 (0.34)	<0.0001
Normal weight, *n* (%)	186 (19.14)	144 (21.83)	42 (14.24)		317 (17.1)	275 (19.2)	42 (10.22)	
Overweight, *n* (%)	550 (55.23)	353 (48.14)	197 (68.18)		550 (33.3)	474 (35.1)	76 (27.2)	
Obesity, *n* (%)	236 (25.62)	186 (30.03)	50 (17.58)		776 (49.6)	592 (45.7)	184 (62.6)	
Diabetes				0.11				0.21
No, *n* (%)	685 (76.58)	502 (78.55)	183 (73.00)		1,088 (71.0)	890 (72.2)	198 (66.9)	
Yes, *n* (%)	287 (23.42)	181 (21.45)	106 (27.00)		555 (29.0)	451 (27.8)	104 (33.1)	
Hypertension				0.01				<0.001
No, *n* (%)	364 (40.66)	283 (44.96)	81 (32.83)		667 (45.2)	579 (48.7)	88 (33.6)	
Yes, *n* (%)	608 (59.34)	400 (55.04)	208 (67.17)		976 (54.8)	762 (51.3)	214 (66.4)	
Smoking status				0.05				0.47
Never, *n* (%)	631 (59.59)	464 (62.80)	167 (53.73)		655 (42.9)	549 (43.1)	106 (42.1)	
Former, *n* (%)	137 (16.12)	96 (16.16)	41 (16.05)		662 (40.7)	516 (39.7)	146 (43.9)	
Now, *n* (%)	204 (24.29)	123 (21.04)	81 (30.21)		326 (16.4)	276 (17.2)	50 (14.0)	
Alcohol status				0.38				0.09
Never, *n* (%)	199 (18.88)	143 (18.56)	56 (19.47)		155 (7.1)	135 (7.8)	20 (4.9)	
Former, *n* (%)	81 (9.66)	55 (10.82)	26 (7.56)		434 (21.1)	341 (20.4)	93 (23.5)	
Mild, *n* (%)	261 (32.45)	172 (30.60)	89 (35.81)		674 (48.3)	540 (46.6)	134 (53.6)	
Moderate, *n* (%)	136 (17.59)	90 (16.54)	46 (19.48)		150 (10.4)	123 (10.7)	27 (9.4)	
Heavy, *n* (%)	295 (21.43)	223 (23.48)	72 (17.68)		230 (13.1)	202 (14.5)	28 (8.6)	
Testosterone (ng/dL), mean (SE)	20.79 (0.58)	21.38 (0.65)	19.73 (0.83)	0.08	408.33 (5.88)	415.18 (5.36)	385.59 (13.90)	0.04
Estradiol (pg/mL), mean (SE)	10.04 (1.08)	11.76 (1.72)	6.91 (0.34)	0.01	25.26 (0.26)	25.06 (0.36)	25.95 (0.83)	0.39
TT/E2 ratio, mean (SE)	0.42 (0.02)	0.44 (0.03)	0.38 (0.02)	0.02	1.74 (0.04)	1.78 (0.05)	1.59 (0.06)	0.01
SHBG (nmol/L), mean (SE)	69.50 (1.65)	69.06 (2.01)	70.32 (2.13)	0.62	53.13 (0.97)	53.36 (1.06)	52.36 (1.85)	0.62
Menopause				0.002				
No, *n* (%)	250 (23.01)	156 (19.42)	94 (29.54)					
Yes, *n* (%)	722 (76.99)	527 (80.58)	195 (70.46)					

All the above mean, standard error, and percentage of frequency were weighted except for the frequency. SE, standard error; PIR, poverty income ratio; BMI, body mass index; SHBG, sex hormone-binding globulin.

### 3.2 Association of testosterone, estradiol, or TT/E2 ratio with OA in males

We examined the association of testosterone, estradiol, and the TT/E2 ratio with OA in males using multivariable logistic regression models to account for potential confounders (Table 2). In multivariate logistic regression, testosterone and estradiol were not associated with OA in all models. Initially, in the crude model (no covariate adjustment) and in Models 1–3 (adjusting for variables like age, race, and lifestyle factors), the TT/E2 ratio was inversely associated with OA risk, suggesting that higher TT/E2 levels might be protective against OA (OR = 0.71, 95% CI = 0.52–0.96, *p* = 0.03). However, when additional covariates were included in Models 4 and 5, including BMI and SHBG, the association was no longer statistically significant. This attenuation suggests that these factors may partially account for the relationship observed in simpler models, indicating potential confounding effects of BMI and SHBG on the TT/E2 ratio’s association with OA.

**Table 2:** Association of testosterone, estradiol, or TT/E2 ratio with OA in males.

	OR (95% CI)	*p*
Testosterone (ng/dL)		
Crude model	1.00 (1.00, 1.00)	0.06
Model 1	1.00 (1.00, 1.00)	0.08
Model 2	1.00 (1.00, 1.00)	0.07
Model 3	1.00 (1.00, 1.00)	0.13
Model 4	1.00 (1.00, 1.00)	0.88
Model 5	1.00 (1.00, 1.00)	0.92
Estradiol (pg/mL)		
Crude model	1.01 (0.99, 1.03)	0.37
Model 1	1.01 (0.99, 1.03)	0.35
Model 2	1.01 (0.99, 1.03)	0.34
Model 3	1.01 (0.98, 1.03)	0.54
Model 4	1.00 (0.98, 1.02)	0.93
Model 5	1.00 (0.98, 1.02)	0.99
TT/E2 ratio		
Crude model	0.71 (0.53, 0.94)	0.02
Model 1	0.72 (0.54, 0.97)	0.03
Model 2	0.71 (0.52, 0.96)	0.03
Model 3	0.77 (0.56, 1.05)	0.09
Model 4	0.98 (0.81, 1.18)	0.81
Model 5	0.99 (0.83, 1.18)	0.90

OR, odds ratio; CI, confidence interval. Crude model: no covariates were adjusted. Model 1: age and race. Model 2: age, race, smoking, and alcohol state. Model 3: age, race, hypertension, DM, smoking, and alcohol state. Model 4: age, race, hypertension, DM, smoking, alcohol state, and BMI. Model 5: age, race, hypertension, DM, smoking, alcohol state, BMI, and SHBG.

**Figure 2:**
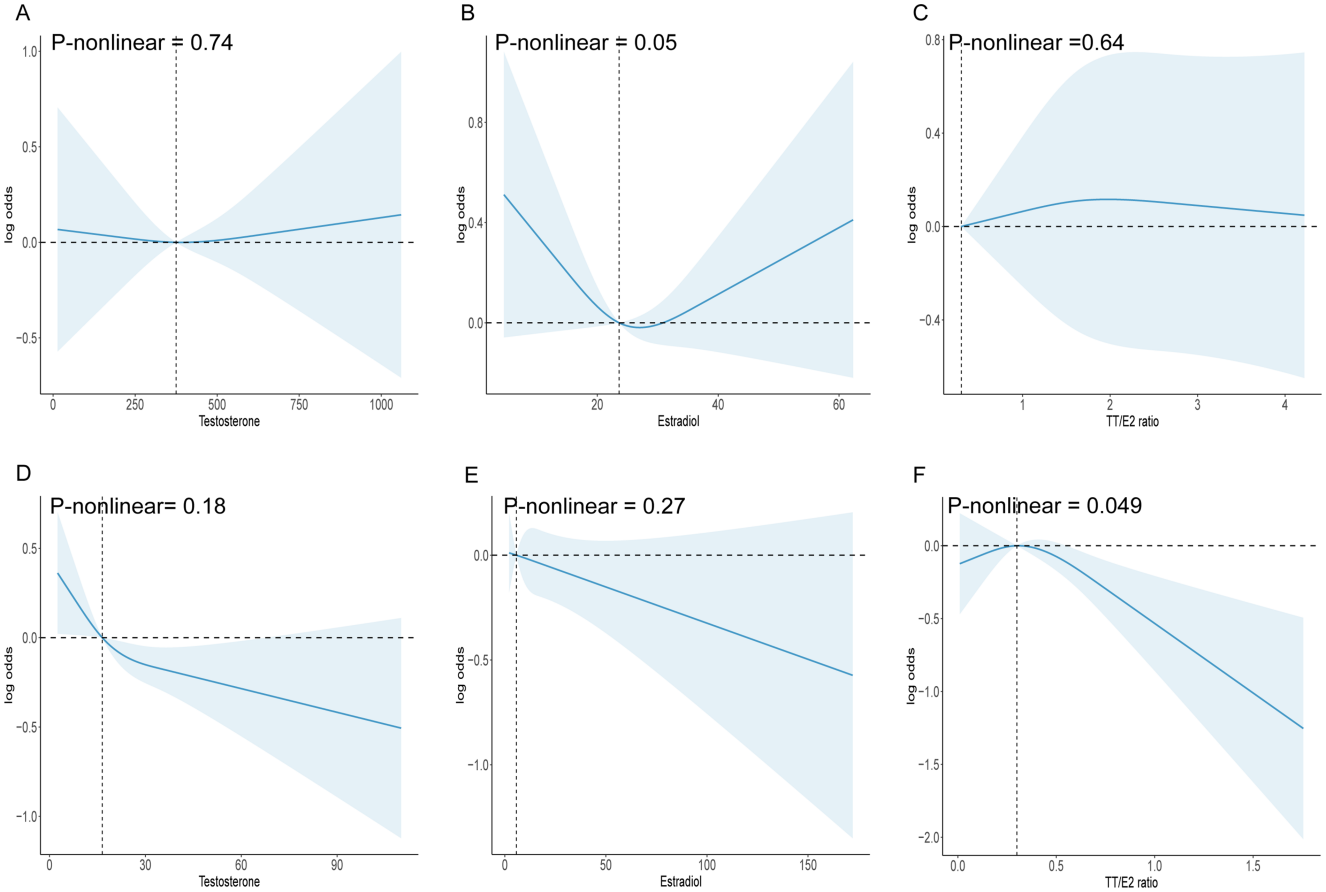
**(A)** RCS model analysis the nonlinear association between testosterone and OA in males. **(B)** RCS model analysis the nonlinear association between estradiol and OA in males. **(C)** RCS model analysis the nonlinear association between TT/E2 ratio and OA in males. **(D)** RCS model analysis the nonlinear association between testosterone and OA in females. **(E)** RCS model analysis the nonlinear association between estradiol and OA in females. **(F)** RCS model analysis the nonlinear association between TT/E2 ratio and OA in females.

**Figure 3:**
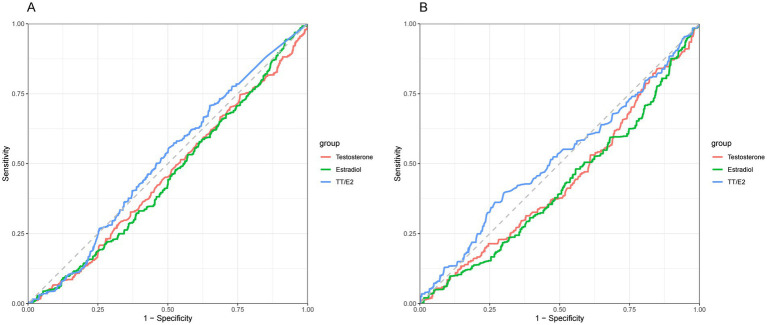
**(A)** ROC analyses of the sex hormones and OA in males. **(B)** ROC analyses of the sex hormones and OA in females.

We applied an RCS model to evaluate potential nonlinear associations. *p*-values for nonlinearity (testosterone *p* = 0.74, estradiol *p* = 0.05, TT/E2 ratio *p* = 0.64) indicated no significant nonlinearity, suggesting a consistent (though nonsignificant) pattern across hormone levels without specific thresholds where the relationship with OA risk changes (Figure 2). The ROC analyses showed that TT/E2 ratio had a favorable performance to predict OA with an area under the curve (AUC) of 0.51 (Figure 3). These findings align with our main analyses, where only marginal effects were observed for sex hormones on OA risk.

### 3.3 Association of testosterone, estradiol, or TT/E2 ratio with OA in females

In females, we also examined the associations between testosterone, estradiol, and TT/E2 ratio with OA through similar logistic regression models. Unlike in males, the TT/E2 ratio maintained a significant inverse association with OA across all models, even when fully adjusted (OR = 0.61, 95% CI = 0.41–0.91, *p* = 0.02). This consistency suggests that in females, the TT/E2 ratio may serve as a more reliable marker of OA risk, independent of BMI, SHBG, and other confounders.

The RCS analysis revealed a significant nonlinear association between the TT/E2 ratio and OA (*p*-nonlinear = 0.049). Specifically, the association became prominent when the TT/E2 ratio exceeded 0.3, suggesting a threshold effect where elevated TT/E2 levels may confer a protective benefit against OA. The ROC analyses showed that TT/E2 ratio had a favorable performance to predict OA with an area under the curve (AUC) of 0.52 (Figure 3) (see Table 3).

**Table 3:** Association of testosterone, estradiol, or TT/E2 ratio with OA in females.

	OR (95% CI)	*p*
Testosterone (ng/dL)		
Crude model	0.99 (0.98, 1.00)	0.08
Model 1	0.99 (0.98, 1.00)	0.12
Model 2	0.99 (0.98, 1.00)	0.15
Model 3	0.99 (0.98, 1.00)	0.19
Model 4	0.99 (0.98, 1.01)	0.25
Model 5	0.99 (0.98, 1.01)	0.23
Model 6	0.99 (0.98, 1.01)	0.25
Estradiol (pg/mL)		
Crude model	1.00 (0.99, 1.00)	0.31
Model 1	1.00 (0.99, 1.00)	0.56
Model 2	1.00 (0.99, 1.00)	0.45
Model 3	1.00 (0.99, 1.00)	0.37
Model 4	1.00 (0.99, 1.00)	0.38
Model 5	1.00 (0.99, 1.00)	0.31
Model 6	1.00 (0.99, 1.00)	0.18
TT/E2 ratio		
Crude model	0.53 (0.39, 0.72)	<0.001
Model 1	0.42 (0.29, 0.59)	<0.0001
Model 2	0.40 (0.28, 0.57)	<0.0001
Model 3	0.43 (0.30, 0.62)	<0.001
Model 4	0.58 (0.40, 0.85)	0.01
Model 5	0.54 (0.36, 0.83)	0.01
Model 6	0.61 (0.41, 0.91)	0.02

OR, odds ratio; CI, confidence interval. Model 1: age and race. Model 2: age, race, smoking, and alcohol state. Model 3: age, race, hypertension, DM, smoking, and alcohol state. Model 4: age, race, hypertension, DM, smoking, alcohol state, and BMI. Model 5: age, race, hypertension, DM, smoking, alcohol state, BMI, and SHBG. Model 6: age, race, hypertension, DM, smoking, alcohol state, BMI, SHBG, and menopause state.

## 4 Discussion

OA is an age- and gender-related degenerative disease. Previous studies have shown that hormone levels, particularly sex hormones, are closely related to the development of OA. However, our study found no significant association between sex hormone levels (testosterone and estradiol) and OA among middle-aged and older individuals in the United States. Our results found that the TT/E2 ratio is independently related to OA, and as the TT/E2 ratio increases, the risk of OA decreases, and this phenomenon is more significant in females, as opposed to the attenuated trend in males, suggests a sex-specific hormonal influence on OA pathogenesis. One plausible justification is that females, particularly postmenopausal, experience substantial changes in hormonal milieu, including decreased estradiol levels, which may amplify the influence of the TT/E2 ratio. Testosterone’s potential chondroprotective effects could also be more pronounced in this population due to hormonal balance shifts (14). Conversely, the lack of a consistent trend in males might reflect a more stable hormonal environment or a reduced sensitivity of male cartilage to TT/E2 variations. Therefore, our current study suggests that the TT/E2 ratio in females may help classify subjects more susceptible to OA independent of traditional risk factors.

We are the first study to explore the relationship between testosterone, estradiol and TT/E2 ratio and OA based on the NHANES database. Similarly, a previous article explored the relationship between testosterone and arthritis, and they found that there was an inverse correlation between testosterone and arthritis, and the relationship was stronger in female and obesity people (BMI ≥30 kg/m^2^). They simultaneously include rheumatoid arthritis, OA and other types of arthritis. But after correcting for more confounding factors, this relationship weakened (15). In our study, we found that testosterone may not be related to the occurrence of OA. Currently, there are some clinical studies proving that testosterone may have a protective effect on OA, but there are also gender differences in this effect. In premenopausal females, lower testosterone level is significantly associated with higher risk of hand OA (16). Freystaetter et al. (14) found that in females, testosterone levels were inversely related to knee function and radiographic knee OA, whereas this relationship did not exist in males. Similarly, Jin et al. (7) found that testosterone was negatively associated with knee pain and effusion-synovitis volume only in females. In summary, testosterone may have a protective effect in female patients with OA. Based on the above evidence, Koelling and Miosge (17) found that testosterone replacement therapy has an improving effect on cartilage in OA. However, there are some studies that do not support the above conclusions. A Mendelian randomization result suggests that serum testosterone levels are positively associated with hip OA and increase the risk of hip replacement (18).

Similar to testosterone, there is currently controversy regarding the role of estradiol in OA. An Australian cohort study of 2,621 females found that lower estradiol concentrations were a risk factor for knee OA (19). Females with a history of hysterectomy had higher risk of radiographic knee OA and first carpometacarpal joint OA (20). Ma et al. (21) found that in ovariectomized female OA mice, the severity of OA was significantly higher than that in the control group. At the same time, exogenous estradiol supplementation can reverse cartilage damage (22). However, there are some studies with conflicting conclusions. In a large Korean cohort study of 1.13 million females, estrogen replacement therapy was associated with a higher risk of knee replacement (23). Similarly, an Australian cohort study also obtained similar results (24).

Our research indicates that it is not testosterone or estradiol individually, but instead their ratio, which exhibits a significant relationship with OA. We discovered that the TT/E2 ratio is inversely related to OA, and this relationship is more consistent in females. At present, the TT/E2 ratio has been used as an indicator to measure the level of male sex hormones, which is significantly correlated with male sexual dysfunction (25), sperm quality (26), cardiovascular disease risk and mortality (9). However, there is a lack of relevant research and potential mechanism of TT/E2 ratio and OA. Recent studies have suggested that this TT/E2 ratio may be associated with the anti-inflammatory protein- soluble forms of glycoprotein130 (sgp130) (27). The sgp130 protein primarily contributes to bind and neutralize IL-6/sIL-6R complexes and inhibit IL-6-trans-signaling. The expression of sgp130 was increased in knee OA chondrocytes, and the release of SGP130 was promoted by inflammatory factors (28). However, there are currently no relevant research reports on the mechanism of sgp130 in OA. Therefore, more high-quality studies are needed to explore the correlation between TT/E2 ratio and OA. Beyond OA, the TT/E2 ratio might have implications in other arthritis types, such as rheumatoid arthritis (RA) or psoriatic arthritis (PsA). Inflammatory pathways like IL-6 trans-signaling, influenced by the TT/E2 balance, could be relevant here as well. For example, studies have demonstrated a link between altered TT/E2 ratios and systemic inflammation in RA patients, where higher testosterone levels correlate with reduced inflammatory markers. Similarly, PsA, characterized by chronic inflammation and enthesitis, might also involve hormonal modulation. Exploring TT/E2’s role could illuminate shared and distinct mechanisms across arthritic conditions (6).

However, our study also has certain limitations. Firstly, it should be noted that this study adopts a cross-sectional design, which restricts the ability to establish a causal relationship between sex hormones and OA. Currently, there are no high-quality randomized controlled studies. Recently, there was a Mendelian randomization result that suggested that testosterone or dihydrotestosterone may be positively related to hip OA and hip replacement (18). Secondly, our definition of OA relies on self-reported arthritis, which may introduce some degree of bias into the results. This study focused on U.S. citizens, and the conclusions may not apply to other populations. In this study, serum testosterone and estradiol levels below the lower limit of LLOD were assigned a value equal to the LLOD divided by the square root of 2. However, this approach prevents missing data from undetectable hormone levels, it introduces limitations in data precision and interpretation. Since the TT/E2 ratio is derived from the measured levels of testosterone and estradiol, the use of substituted values below LLOD could lead to inaccuracies, particularly when either hormone is undetectable. This could affect the interpretation of the ratio’s relationship with OA prevalence, especially in populations with generally lower hormone levels, such as postmenopausal female. Future studies could improve accuracy by employing more sensitive detection techniques or using multiple imputation methods to estimate values below the LLOD more dynamically. Finally, we did not consider the interference of menopause in the multivariate logistic regression analysis. Since the people we include are mainly over 50 years old, most females in this age group are already menopausal. The study ultimately enrolled 3,231 participants, with the exclusion of 2,374 individuals aged over 50 who did not meet the inclusion criteria (response rate achieved was 57.6%). Due to a significant proportion of patients having missing hormone data, it was unfeasible to establish a correlation between hormone levels and the prevalence of OA within these cohorts.

In summary, our findings indicate an inverse association between the TT/E2 ratio and OA, particularly among females. These results offer potential insights for identifying risk factors associated with OA. However, future longitudinal studies of higher methodological rigor are warranted to elucidate the causal relationship between the TT/E2 ratio and OA.

## Data Availability

The original contributions presented in the study are included in the article/supplementary material, further inquiries can be directed to the corresponding author.

## Footnotes

^1^ https://www.cdc.gov/nchs/nhanes/index.htm
